# Vitamin B2 and Folate Concentrations are Associated with ARA, EPA and DHA Fatty Acids in Red Blood Cells of Brazilian Children and Adolescents

**DOI:** 10.3390/nu11122918

**Published:** 2019-12-02

**Authors:** Fábio V. Ued, Mariana G. Mathias, Roseli B. D. Toffano, Tamiris T. Barros, Maria Olímpia R. V. Almada, Roberta G. Salomão, Carolina A. Coelho-Landell, Elaine Hillesheim, Joyce M. Camarneiro, José Simon Camelo-Junior, Davi C. Aragon, Sofia Moco, Martin Kussmann, Jim Kaput, Jacqueline P. Monteiro

**Affiliations:** 1 Department of Pediatrics, Ribeirão Preto Medical School, University of São Paulo, Bandeirantes Avenue, 3900, Ribeirão Preto 14049-900, Brazil; marimathias@hotmail.com (M.G.M.); roselibdt@hotmail.com (R.B.D.T.); tamiris.barros.7@gmail.com (T.T.B.); madovale@yahoo.com.br (M.O.R.V.A.); rsalomao@usp.br (R.G.S.); k_rolacoelho@hotmail.com (C.A.C.-L.); elainehillesheim@usp.br (E.H.); joycecamarneiro@gmail.com (J.M.C.); jscamelo@fmrp.usp.br (J.S.C.-J.); dcaragon@fmrp.usp.br (D.C.A.); jacque@fmrp.usp.br (J.P.M.); 2 Department of Health Sciences, Ribeirão Preto Medical School, University of São Paulo, Bandeirantes Avenue, 3900, Ribeirão Preto 14049-900, Brazil; 3 Nestlé Institute of Health Sciences, École Polytechnique Fédérale de Lausanne, Campus, Innovation Square, 1015 Lausanne, Switzerland; sofia.moco@rd.nestle.com (S.M.); m.kussmann@auckland.ac.nz (M.K.); jkaput@gmail.com (J.K.)

**Keywords:** fatty acids, B-vitamins, homocysteine, child, adolescent

## Abstract

Vitamins B2, B6, B12, and folate are essential for methylation reactions and possibly influence the transport of polyunsaturated fatty acids in plasma and red blood cells (RBC). Associations between B-vitamin biomarkers and fatty acid (FA) profile were analyzed in Brazilian children and adolescents. This cross-sectional study included 249 children and adolescents, aged 9–13 years old. Dietary intake was assessed by the food frequency questionnaire and the healthy eating index (HEI). Biomarkers for vitamins B2, B6, B12, and folate were measured in plasma. The FA profile and the metabolites of one-carbon metabolism were measured in RBC. Associations were tested with multiple linear regression models. An increase of 1 nmol/L in vitamin B2 was associated with an increase of 0.19 mg/dL of EPA, 0.20 mg/dL of ARA, and 0.25 mg/dL of DHA in RBC. An increase of 1 ng/mL in plasma folate was associated with an increase of 0.14 mg/dL of EPA, 0.22 mg/dL of ARA, and 0.21 mg/dL of DHA in RBC. These findings highlight the importance of an adequate intake of vitamin B2 and folate in childhood, since they may improve the FA profile in RBCs and may help prevent cardiovascular disease.

## 1. Introduction

Cardiovascular risk factors have become prevalent in children and adolescents [1,2]. The prevalence of dyslipidemia, hypertension, insulin resistance, and excess body fat, in individuals with excess weight or appropriate weight, have been increasing [3,4,5]. The main causes are attributed to sedentary lifestyles and unbalanced diets in childhood [5].

Cardiovascular diseases (CVD) are among the leading causes of morbidity and mortality worldwide, with atherosclerosis as the principal cause of death [6]. Evidence suggests that the process of atherosclerosis begins in childhood [7] and progresses through adulthood [8] depending on genetic susceptibility influenced by nutrition, lifestyle, and environmental factors.

Dyslipidemia is a common cardiovascular risk factor in childhood, defined by changes in serum or erythrocyte lipid and fatty acid (FA) levels [9,10]. Altered lipid levels are related to atherosclerotic lesions in the pediatric population [11,12]. Longitudinal studies have shown that nutritional interventions in children are effective for the prevention of CVD in adults [5,8].

Total homocysteine (tHcy) concentration is also considered as a CVD risk factor [13] in childhood due to its role in oxidative stress, endothelial dysfunction, and thrombogenicity enhancement [14]. A debate exists, however, about whether tHcy is a biomarker of effect [15] or an independent risk factor [16] for CVD. Results of a recent meta-analysis show that interventions to reduce tHcy levels did not prevent cardiovascular events [17]. The positive associations between tHcy levels and CVD can be confounded by the presence of other cardiovascular risk factors [15], such as low levels of n-3 polyunsaturated fatty acids (PUFAs) [18,19].

Homocysteine is a part of one-carbon (1C) metabolism. Vitamins B2, B6, B12, and folate participate in this pathway and influence the production of S-adenosylmethionine (SAM), the primary methyl (CH3) group donor for proteins, DNA, and RNA, and transferred in many metabolic reactions [20]. For example, methylation is involved in the conversion of phosphatidylethanolamine (PE) to phosphatidylcholine (PC). PC is essential for the transport of PUFAs in plasma and red blood cells (RBCs) [21,22]. Therefore, B-vitamins may help prevent CVD by at least two processes: (i) reducing tHcy levels; and (ii) interfering with the methylation process of PE to PC, influencing the concentrations of PUFAs in plasma and in RBCs.

The interactions between B-vitamins and the FA remains poorly understood. Studies in animal models [22,23,24,25] and humans [26,27,28,29,30] have produced conflicting results. Few reports have been published analyzing these associations in late childhood and early adolescence [18]. The aim of this study was to examine the association between FAs, clinical lipid profile, tHcy levels, and B-vitamins biomarkers in healthy Brazilian children and adolescents.

## 2. Materials and Methods

### 2.1. Study Design

The data described in this cross-sectional study were from the crossover N-of-1 micronutrient intervention study previously reported [31]. Briefly, a six-week multivitamin/mineral intervention was conducted in 9–13 year olds. Participants were: (i) their own control (N-of-1); (ii) monitored for compliance; and (iii) measured for food intake, anthropometric and metabolites in plasma and RBCs, at baseline (Visit 1), post intervention (Visit 2), and following a 6-week washout (Visit 3) in two consecutive years, 2013 and 2014 [31]. To avoid the influence of these supplements on plasma and RBC metabolites, only the baseline data (Visit 1) were used in the analyses described here.

Data collection was performed at the Ribeirão Preto Medical School Hospital (HCFMRP-USP), University of São Paulo, Brazil. The study was approved by the internal ethics committee (Process HCRP No. 14255/2010) and by the National Research Ethics Commission (No. 00969412.6 CAAE. 0000.5440). The trial was registered on ClinicalTrials.gov (NCT01823744). The participants were informed about the purpose and procedures of the study and signed a statement of informed consent. Parents of each participant signed informed consent.

### 2.2. Population

Participants in this study were clinically stable children and adolescents, i.e., without injury, chronic noncommunicable diseases, or infectious diseases, as specified in exclusion criteria. Children and adolescents aged 9–13 years, were recruited from three schools in the west side of Ribeirão Preto. This municipality is in the northeastern region of the state of São Paulo in Brazil. 

Exclusion criteria were individuals: (i) with one or more episodes of axillary temperature higher than 37 °C in the 15 days preceding the blood collection; (ii) with three or more episodes of liquid stools in the 24 h before assessment; (iii) with intake of any kind of vitamin or mineral supplement; (iv) on a supervised diet for reducing weight or any other type of dietary restriction; (v) with a diagnosis of chronic disease that may interfere with data collection; and (vi) who participated in another clinical trial in the four weeks preceding the study.

The upper age cut-off was 13 years, 11 months and 29 days at registration visit (Visit 1). Individuals in all weight groups were included. A total of 280 participants met the inclusion criteria. After removing siblings and outliers of clinical, vitamin, and FA levels, 249 participants were considered for analysis.

### 2.3. Data Collection

All participants were assessed for anthropometric, pubertal [32], and economic status [33]. Physical activity and dietary intake were also evaluated. A dietitian measured height and weight of participants immediately after fasted blood collection (12 h) using the procedures described by Jellife [34] and the World Health Organization (WHO). Body mass index (BMI) was calculated according to WHO [35] and used for nutritional status classification. Blood was collected in EDTA tubes for biochemical analysis in plasma and RBC. All samples were coded at the time of collection, centrifuged, aliquoted, and frozen at −80 °C for further analyses.

### 2.4. Physical Activity Assessment

The level of physical activity was evaluated during three days using the BodyMedia (now Jawbone, Pittsburgh, PA, USA) Sensewear^®^, an activity and caloric expenditure measuring device. The arm band incorporated sensors for galvanic skin response, skin temperature, heat flux, and two axis accelerometery with proprietary algorithms to calculate metabolic equivalent (MET) [36]. The level of physical activity for a school day was classified as sedentary, mild, moderate, or vigorous according to the MET classification [37].

### 2.5. Dietary Assessment

Current dietary intake was assessed by 24-h recalls. One parent or guardian was also present for the assessment. The Healthy Eating Index (HEI) was computed from data obtained from three nonconsecutive 24-h recalls [31], including a weekend day. The HEI used in this study was previously revised for the Brazilian population [38] and subsequently validated for children and adolescents [39].

The HEI is estimated by scoring 12 components that characterize different aspects of a healthy diet [38]. Each component is evaluated and scored from a minimum of 0 to a maximum of 20. The first nine components of the HEI are food groups. Total saturated fat, sodium, and calories from solid fat, alcohol, and added sugar constitute the other three components and are scored in the opposite direction to the other components (i.e., lower intakes have higher scores). For all components based on food groups, a full score is given for intakes at or above recommended amounts. A zero indicates that no foods in that group were consumed, whereas intermediate numbers of servings are awarded prorated scores. The maximum HEI score is 100 [38].

Usual dietary intake was assessed by the adapted Brazilian Food Frequency Questionnaire (FFQ) [40]. The children and adolescents and their legal representatives were interviewed about frequency of food intake and portion sizes during the previous month using the photographic manual adapted by Monteiro et al. [41]. The estimated average requirement (EAR) and the adequate intake (AI) from Dietary Reference Intake (DRI) were used to determine if the population nutrient intake was adequate [42,43]. The data reported from the FFQ were transformed into daily frequencies according to Araújo et al. [44]. These scores were multiplied by the size of food portion consumed by the participant.

Intakes of energy, carbohydrates, proteins, lipids, cholesterol, omega-3, omega-6, and B-vitamins (B2, B6, B12, and folic acid) were determined. Diet data were double-checked during transfer into the DietWin Professional software version 2011 (Dietwin Software de Nutrição, Porto Alegre, Brazil), which was used for analyzing dietary intake data. This program includes 5000 food items from six food composition databases (Tabela Brasileira de Composição de Alimentos, Instituto Brasileiro de Geografia e Estatística, U.S. Department of Agriculture, Centro de Endocrinología Experimental y Aplicada, and General Directory of Food) and more than 1300 recipes.

### 2.6. Laboratory Analyses

Blood samples were centrifuged to separate RBCs from plasma. Plasma lipid profile (total cholesterol, triglycerides, LDL-c, and HDL-c) was determined in plasma by an enzymatic method, and the analysis was conducted immediately after the blood draw in the HCFMRP-USP laboratory using standard procedures on a Wiener Lab CT 600i analyzer (Diamond Diagnostics, Holliston, MA, USA). Samples used for the analysis of other metabolites (FA, B-vitamin biomarkers and homocysteine cycle metabolites) were frozen at −80 °C and transferred to the Nestlé Institute of Health Sciences, Nestlé Research Center laboratories (Lausanne, Switzerland), and Vitas Analytical Services (Oslo, Norway).

The analysis of metabolites investigated in this study has already been described by Mathias et al. [31]. Briefly, FA were measured in RBC (200 μL RBC lysed with 200 μL of NH4Cl 82.29 mg/mL, NaHCO3 10.00 mg/mL, and EDTA 292.2 mg/mL) and analyzed by gas–liquid chromatography (GLC) [45,46]. The main saturated fatty acids (SFAs), monounsaturated fatty acids (MUFAs), and PUFAs were measured in RBC. The levels of tHcy, methionine, SAM, S-adenosylhomocysteine (SAH), cystathionine, and cysteine were measured in RBC by liquid chromatography tandem mass spectrometry (LC-MS/MS) [47]. These metabolites were measured in RBC because SAM and SAH levels are higher in erythrocytes [48,49,50,51,52] and may be less affected by physiological processes that induce cell death or turnover [48]. RBC appear to contain all SAM found in the blood [49] since SAM is metabolized in these cells, and are excellent indicators of the level of methyl availability in animal tissues [49]. Riboflavin, vitamin B2 subclass, pyridoxal, pyridoxal 5’-phosphate (PLP), vitamin B6 subclass, and 5-methyl tetrahydrofolate (5-MTHF) were analyzed in plasma by LC-MS/MS [53]. Plasma vitamin B12 and plasma folate were analyzed through AM-396 and MonoBind ELISA (Folate/Vitamin B12 Anemia Panel VAST test system, Monobind, Lake Forest, CA, USA). The headings “SFA”, “MUFA”, “PUFA”, “vitamin B2 subclass”, and “vitamin B6 subclass” include all biomarkers of these FA and vitamins (respectively) identified during blood analysis.

### 2.7. Statistical Analyses

The sample size was determined by the feasibility of recruitment. Considering that the main association of the study is between the levels of DHA and vitamin B2 subclass, the effect size (Cohen’s ƒ2) [54] is equal to 0.16, which can be assumed as a moderate effect (*f*2 ≥ 0.15).

Outliers were identified using principal component analysis for clinical, vitamin, and FA parameters [31]. Outliers were defined as samples falling outside of the 99% confidence ellipse. Six individuals were identified as statistical outliers for clinical variables and circulating vitamin levels. Seven individuals were statistical outliers for FA profile. Eighteen individuals were identified as siblings. These subjects were excluded from further analyses.

Population characteristics are presented as means, standard deviation, and median (1st quartile–3rd quartile). Tests for normality were performed using the Kolmogorov–Smirnov test. B-vitamin biomarkers, fasting lipids, RBC fatty acids, and tHcy-related variables were logarithmically transformed to obtain a normal distribution.

Associations among levels of lipid, FAs, tHcy, and B-vitamin (see Table 1 for references and range of values) biomarkers were tested with multiple linear regression models, adjusting for sex, pubertal staging, BMI, and HEI. Positive β-coefficients unstandardized indicate positive association, while negative β-coefficients unstandardized indicate negative association, with a *p*-Value < 0.05 as the significant cutoff. All statistical analyses were performed using the Statistical Package for the Social Sciences (SPSS), version 20.0 (IBM, New York, NY, USA).

## 3. Results

After removing statistical outliers and siblings, 249 children and adolescents (55.4% female) participated in the study. The average age was 11.6 ± 1.1 years with 19 individuals (7.6%) in pubertal stage 1, 84 (33.7%) in stage 2, 100 (40.2%) in stage 3, 40 (16.1%) in stage 4, and 6 (2.4%) in stage 5. Based on the classification of BMI for age, seven participants (2.8%) were severely thin, 24 (9.6%) thin, 110 (44.2%) at the appropriate weight, 55 (22.1%) overweight, and 53 (21.3%) were obese. The majority of the participants belonged to category B2 (35.3%—monthly family income of US$812.00) and C1 (28.1%—monthly family income of US$494.00) socioeconomic classes. Two hundred twelve children and adolescents (85.1%) were classified into levels of mild and sedentary physical activity. Physical activity did not change the analysis of association among levels of lipid, FAs, tHcy, and B-vitamins biomarkers (data not shown), therefore was not considered for further analyses.

Dietary analysis showed high inadequacy of omega-3 and folate intakes. The HEI total score showed low diet quality, with low intake of vegetables, fruits, and whole grains. The circulating levels of most vitamins were below normal ranges for a pediatric and adolescent population based on best available reference values (Table 1). Associations presented in Table 2 show that no B-vitamin biomarker was associated with a better lipid profile, i.e., high levels of HDL-c and low levels of total cholesterol, triglycerides, or LDL-c. Vitamin B2 and B6 were positively associated with total cholesterol and LDL-c. An increase of 1 nmol/L in vitamin B2 was associated with an increase of 0.05 mg/dL of total cholesterol and 0.09 mg/dL of LDL-c.

Overall, significant associations were found between B-vitamins biomarkers and the FAs (Table 3). Vitamin B2 and plasma folate showed positive associations with linoleic acid (LA), α-linolenic acid (ALA), arachidonic acid (ARA), eicosapentaenoic acid (EPA), and docosahexaenoic acid (DHA). An increase of 1 nmol/L in vitamin B2 was associated with an increase of 0.15 mg/dL of LA and ALA, 0.19 mg/dL of EPA, 0.20 mg/dL of ARA, and 0.25 mg/dL DHA. An increase of 1 ng/mL in plasma folate was associated with an increase of 0.15 mg/dL of LA and ALA, 0.14 mg/dL of EPA, 0.22 mg/dL of ARA, and 0.21 mg/dL of DHA.

Vitamin B2 also had a positive association with MUFAs such as palmitoleic and oleic acid, and negative association with elaidic *trans*-FA. Vitamin B12 was positively associated with MUFAs (palmitoleic and oleic acid) and SFAs (palmitic, stearic and eicosanoic acid). In contrast, vitamin B6 was negatively associated with the PUFAs LA, ALA, ARA, and DHA.

Vitamin B2 was inversely associated with the concentrations of the 1C metabolites SAM and the SAM/SAH ratio (Table 4). An increase of 1 nmol/L in vitamin B2 was associated with a reduction of 0.18 μmol/L of SAM and 0.20 points in the SAM/SAH ratio. Only vitamin B6 and B12 were associated with increased SAM/SAH ratio and lower tHcy levels. An increase of 1 nmol/L in vitamin B6 and 1 pg/mL in plasma vitamin B12 was associated with a reduction of 0.11 μmol/L and 0.14 μmol/L of tHcy, respectively. The associations between FAs, clinical lipid profile, tHcy levels, and other B-vitamins biomarkers are shown in Supplementary Materials (Tables S1–S3). In addition, tHcy levels were positively associated with PUFAs concentrations (Table S4). An increase of 1 μmol/L in tHcy was associated with an increase of 0.24 mg/dL of LA and ALA, 0.38 mg/dL of ARA, 0.35 mg/dL of EPA, and 0.49 mg/dL of DHA.

## 4. Discussion

To our knowledge, this is the first study to investigate the association between FAs, lipid profiles, tHcy levels, and B-vitamins biomarkers in healthy children and adolescents, aged 9–13 years. Our results show that higher concentrations of vitamin B2 and plasma folate were associated with higher RBC levels of LA, ALA, ARA, EPA, and DHA. Vitamin B6 and B12 were associated with lower tHcy levels but were not associated with higher levels of PUFAs. No B-vitamin biomarker was associated with a lipid profiles considered to be healthy (low TG, LDL-c, and high HDL-c).

The associations found in this study were adjusted for HEI scores to attenuate the effect of diet in observed outcomes. Iglesia et al. [18] also used HEI as a covariate to control for diet instead of just FA intake. The association between FA intake and plasma FA cannot be assumed, due to the different pathways that metabolize FA in the human body [61]. Others showed that diet intake of n-3 FA explained only 14.2% of plasma FA concentrations in adolescents [61]. HEI data consider dietary complexity [38] and are used as a confounding variable in statistical analysis. The total HEI score and BMI classification were similar among all economic categories indicating that Brazilian children and adolescents were malnourished and were eating poorly, regardless of family income.

**Figure 1:**
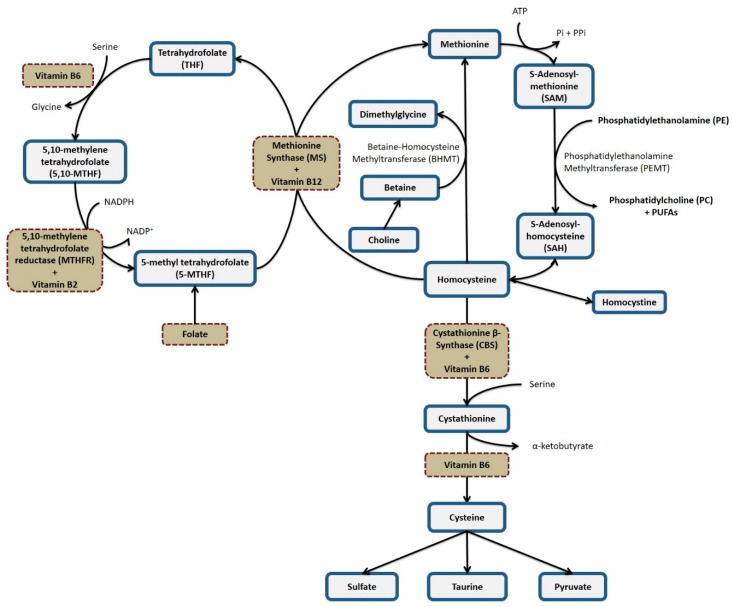
One-carbon metabolism.

Evidence of associations between B-vitamins and FAs is unclear and often debatable. Only one study found this association in adolescents aged 12.5–17.5 years [18]. Studies in adults also demonstrated associations [26,27,30], while studies in elderly did not find any associations [28,29]. Two physiological pathways (see below) may explain the effect of differing vitamin intakes on FA levels [22,30]:

(i) Vitamins B2, B6, B12, and folate act as cofactors in the 1C metabolism and may influence the concentrations of SAM (Figure 1). SAM is used in a variety of transmethylation reactions, one of which is catalyzed by phosphatidylethanolamine methyltransferase (PEMT). This enzyme catalyzes three consecutive methylation reactions, resulting in the conversion of PE to PC [21,22]. PE and PC are fundamental phospholipids for cell membranes [62] and PC side-chains may be enriched in PUFAs [63]. In addition, PC constitutes the majority of plasma phospholipids [64] and is crucial for the transport of PUFAs (especially DHA) from the liver to extrahepatic tissues [21,22]. Hence, B-vitamins influence PE to PC and the concentrations of PUFAs in plasma or RBCs by altering SAM concentrations.

(ii) A second plausible pathway depends on the activity of the enzyme Δ6 desaturase [30]. Preliminary studies have shown reduced activity of the Δ6 desaturase enzyme in vitamin B6 deficiency [65,66], which may produce low concentrations of ARA, EPA, and DHA in plasma, and increase in LA and ALA [66,67]. An inverse association between vitamin B6 levels and concentrations of LA and ALA was found in our study but not with an expected concomitant change in ARA and DHA levels.

Vitamin B2 and plasma folate were the only vitamins that showed positive associations with RBC levels of PUFAs, which may best be explained by changes in 1C metabolism. Vitamin B2 was inversely associated with SAM levels and SAM/SAH. Others found that B-vitamins did not influence plasma SAH concentrations, but did alter plasma tHcy concentrations [68,69,70,71]. An inverse association between plasma vitamin B2 and folate with RBC tHcy levels was not found in our study.

In addition, no inverse association between tHcy and PUFAs levels were detected. tHcy and SAH levels have been found to be inversely associated to PUFAs concentrations [18,22,26]. High levels of SAH may inhibit PEMT and consequently reduce levels of PC and PUFAs in plasma or erythrocytes [22,72]. The inverse associations between tHcy and PUFAs were observed in animals [22] and humans [18,26,72] with tHcy close to or above 10 μmol/L. The level of tHcy in our population was 2.7 μmol/L.

However, tHcy and SAH levels were positively associated with PUFA levels, which may occur since RBCs were used in this study versus plasma fractions in other studies. Our results are consistent with low levels of tHcy associated with the observed levels of SAM and SAH. That is, SAH levels were not in the concentrations needed to inhibit PEMT. Reference values for SAH may be determined in whole blood lysates, serum, plasma, or RBC lysates making it challenging to compare results.

Vitamin B2 in the form of flavin adenine dinucleotide (FAD) is a cofactor for the enzyme 5, 10-methylenetetrahydrofolate reductase (MTHFR) [73], a key reaction in 1C metabolism. Folate is considered the major determinant of tHcy concentrations, compared to vitamins B2, B6, and B12 [74,75]. Others found that vitamin B2 supplementation alters tHcy only in severe cases of hyperhomocysteinemia in individuals homozygous with the C677T polymorphism of the MTHFR gene [74,76] who also have insufficient folate intake [77]. Results in this study associating plasma vitamin B2 and RBC PUFA are consistent with the involvement of the 1C pathway since the population in this study had high inadequacy of folate intake.

Vitamin B2 may also alter FA levels through other metabolic pathways such as the beta-oxidation, since FAD is a cofactor of the enzyme acyl-CoA dehydrogenase. Vitamin B2 supplementation potentiated the beta-oxidation process [78,79], which catalyzes tetracosahexaenoic acid (24:6) to DHA (22:6). This mechanism would explain the interaction between vitamin B2 and DHA. Vitamin B2 was positively associated with levels of n-3 FA, n-6 FA, total cholesterol, and LDL-c. Further studies are needed to determine whether vitamin B2 contributes to a less atherogenic lipid and FA profiles.

Folate and vitamin B12 are the main vitamins associated with higher concentrations of EPA and DHA [18], possibly through their involvement in remethylation steps in the 1C pathway. Positive associations with n-3 FA were found more frequently than associations with n-6 FA [18,23]. In the present study, plasma folate was positively associated with RBC n-3 and n-6 PUFA levels, while vitamin B12 was mainly associated with SFA levels. Intake of animal source proteins have been associated with high SFA [80] consistent with the dietary recall data in this study.

Evidence suggests that n-3 FAs are cardioprotective, whereas excess of n-6 FA is considered to increase the inflammatory potential [81]. No association of B-vitamins with n-6:n-3 pro-inflammatory index was observed, indicating that an increase in B-vitamin levels is associated with a proportional increase in n-3 and n-6. The balance of these PUFAs in childhood plays a key role in the functioning and development of the brain and central nervous system [82].

Different concentrations of B-vitamins may alter FA transport, which in turn can affect kidney health. Folate has been shown to improve renal endothelial function, regardless of the reduction in tHcy levels [83], possibly by influencing the transport of FA in the blood. FA oxidation is the preferred energy source for kidney tubular epithelial cells which may prevent the development of renal fibrosis [84].

B-vitamins are also cofactors in the metabolism of sulfur-containing amino acids, such as methionine, homocysteine, cysteine, and taurine. These amino acids in turn have been reported to influence the activity of the stearoyl-CoA desaturase (Δ9 desaturase) [85,86], a FAD containing enzyme [87] essential for the conversion of palmitic acid (C16:0) to palmitoleic acid (C16:1) and stearic acid (C18:0) in oleic acid (C18:1). These mechanisms may explain the association between vitamin B2 and RBC MUFAs, although no association was found between vitamin B2 and sulfur containing amino acids.

Our data suggest that this population had hidden hunger [88], defined as sufficient energy intake but insufficient consumption of micronutrients. Poor diet quality and overweight have the potential to affect endothelial integrity and trigger vascular damage over time [7,8]. Since dietary intake of B-vitamins [89] and n-3 FA [90] is usually poor in children and adolescents, eating habits in the pediatric population need to be periodically evaluated.

### Strengths and Limitations of the Study

The strengths of this study were: (i) the use of standardized and validated tools for assessing the quality of diet and food intake for Brazilian children and adolescents; (ii) analysis of all B-vitamins involved in the 1C metabolism and its main biomarkers (riboflavin, vitamin B2 subclass, pyridoxal, PLP, vitamin B6 subclass, 5-MTHF, plasma folate, and plasma vitamin B12), which better reflect B-vitamin status and complement dietary assessment methods; and (iii) the measurement of the main metabolites of 1C metabolism (methionine, SAM, SAH, tHcy, cystathionine, and cysteine), which are essential for understanding the mechanism of interaction between B-vitamins and the FAs.

The limitations of this study were: (i) the small sample size, which makes it difficult to extrapolate the results to the entire population of children and adolescents; (ii) the cross-sectional design of the study, which does not allow us to establish cause–effect relationships; (iii) the amount of folic acid contained in fortified wheat flour which is present in foods such as breads, pastas and biscuits, was not recorded in food intake analysis, underestimating the folate intake of children and adolescents; (iv) important indicators of vitamin B12 status, such as levels of methylmalonic acid and holotranscobalamin (the fraction available for tissue uptake), were not measured in plasma; and (v) choline, which might also influence our results, and concentrations of PE and PC were not measured in plasma or RBCs.

## 5. Conclusions

Biomarkers related to vitamin B2 and folate were positively associated with ARA, EPA, and DHA concentrations. The most plausible metabolic pathway to explain such associations is 1C metabolism via increased flux of SAM. However, these vitamins were not associated with higher levels of SAM and lower levels of tHcy. The population studied here already had low baseline tHcy levels blunting the anticipated effect of increased vitamin intake on this metabolite level. Vitamin B2 was also positively associated with total cholesterol and LDL-c, indicating the need for new studies to elucidate the mechanism of interaction of vitamin B2 with the metabolism of lipoproteins and FA.

These findings highlight the importance of ensuring an adequate intake of vitamin B2 and folate since they may improve the FA profile. Appropriate concentrations of n-3 and n-6 FA contribute to the development of central nervous system and renal health and to the prevention of CVD, which are increasingly common due to sedentary lifestyle, poor diet, and excess body fat in children and adolescents. The prevention of CVD risk factors should begin even in childhood, since the habits of life are formed at this stage.

## Figures and Tables

**Table 1:** Baseline clinical and dietary characteristics of participants.

Characteristics	Mean (SD)	Median	Q1–Q3	Reference Value	Inadequacy
Age (years) (249) ^a^	11.6 (1.1)	12	11–13	-	-
BMI (kg/m^2^) (249) ^a^	20.4 (4.8)	19.4	16.5–22.9	-	-
Physical activity (MET) (249) ^a^	2.5 (0.5)	2.4	2.1–2.8	-	-
**Dietary Intake (FFQ)**					
Energy (kcal/day) (249) ^a^	1948.5 (766.8)	1787.0	1389.0–2351.0	- ^b^	-
Carbohydrate (% kcal) (249) ^a^	54.5 (6.2)	54.9	50.7–58.2	45–65% kcal [43]	11.6%
Protein (% kcal) (249) ^a^	14.6 (3.3)	14.2	12.3–16.4	10–30% kcal [43]	5.2%
Lipid (% kcal) (249) ^a^	30.8 (4.7)	30.7	27.9–33.9	25–35% kcal [43]	28.9%
Saturated fat (g/day) (249) ^a^	20.3 (9.6)	18.5	13.6–25.1	- ^b^	-
Cholesterol (mg/day) (249) ^a^	214.8 (111.6)	202.7	134.0–272.3	- ^b^	-
Omega 3 (% kcal) (249) ^a^	0.5 (0.2)	0.5	0.3–0.6	0.6–1.2% kcal [43]	76.7%
Omega 6 (% kcal) (249) ^a^	4.6 (1.9)	4.3	3.3–5.8	5–10% kcal [43]	65.9%
Vitamin B2 (mg/day) (249) ^a^	1.6 (0.8)	1.4	1.0–2.0	0.8 [42]	13.3%
Vitamin B6 (mg/day) (249) ^a^	1.3 (0.6)	1.2	0.9–1.6	0.8 [42]	18.5%
Vitamin B12 (µg/day) (249) ^a^	2.8 (1.5)	2.6	1.7–3.7	1.5 [42]	17.3%
Folate (µg/day) (249) ^a^	105.0 (74.2)	91.4	60.9–126.7	250 [42]	96.8%
HEI total score (249) ^a^	54.8 (10.8)	53.8	47.1–62.5	- ^b^	-
**Lipid Profile (Laboratory)**					
Total cholesterol (mg/dL) (249) ^a^	163.7 (28.8)	163.0	141.5–184.0	<170 [55]	41.8%
Triglycerides (mg/dL) (249) ^a^	71.3 (35.3)	64.0	47.0–85.0	<75 (9 years) [55]<90 (10–13 years) [55]	22.1%
LDL-c (mg/dL) (249) ^a^	103.4 (25.4)	102.0	84.5–120.0	<110 [55]	37.3%
HDL-c (mg/dL) (249) ^a^	46.0 (9.4)	45.0	39.0–52.0	>45 [55]	45.0%
**Fatty Acid Profile (Laboratory)**					
15:0 pentadecanoic (mg/dL) (244) ^a^	0.26 (0.35)	0.20	0.17–0.23	- ^b^	-
16:0 palmitic (mg/dL) (244) ^a^	32.13 (2.95)	31.99	30.56–33.48	- ^b^	-
16:1 (n-7) palmitoleic (mg/dL) (244) ^a^	0.50 (0.14)	0.48	0.40–0.59	- ^b^	-
18:0 stearic (mg/dL) (244) ^a^	25.90 (2.31)	25.83	24.66–27.01	- ^b^	-
18:1 (*trans*) elaidic (mg/dL) (244) ^a^	0.95 (0.23)	0.91	0.79–1.08	- ^b^	-
18:1 (n-9) oleic (mg/dL) (244) ^a^	15.32 (2.20)	15.28	14.08–16.50	- ^b^	-
18:2 (n-6) linoleic (mg/dL) (244) ^a^	15.03 (4.31)	15.39	11.62–18.27	- ^b^	-
18:3 (n-3) α−linolenic (mg/dL) (244) ^a^	0.21 (0.07)	0.20	0.15–0.26	- ^b^	-
20:0 eicosanoic (mg/dL) (244) ^a^	0.70 (0.10)	0.69	0.63–0.77	- ^b^	-
20:4 (n-6) ARA (mg/dL) (244) ^a^	17.41 (7.07)	18.00	10.51–23.55	- ^b^	-
20:5 (n-3) EPA (mg/dL) (244) ^a^	0.40 (0.20)	0.37	0.26–0.52	- ^b^	-
22:6 (n-3) DHA (mg/dL) (244) ^a^	3.97 (2.07)	3.70	2.11–5.61	- ^b^	-
SFA (mg/dL) (244) ^a^	72.08 (6.35)	71.85	68.83–74.95	- ^b^	-
MUFA (mg/dL) (244) ^a^	23.65 (3.44)	23.47	21.34–25.83	- ^b^	-
PUFA (mg/dL) (244) ^a^	39.30 (13.77)	40.36	26.72–51.59	- ^b^	-
n-3 PUFA (mg/dL) (244) ^a^	4.59 (2.27)	4.22	2.53–6.47	- ^b^	-
n-6 PUFA (mg/dL) (244) ^a^	30.70 (11.77)	35.79	23.99–45.15	- ^b^	-
Ratio n-6:n-3 PUFA (244) ^a^	7.81 (2.08)	7.63	6.42–8.94	- ^b^	-
**Homocysteine and Metabolites (Laboratory)**					
Methionine (μmol/L) (194) ^a^	23.66 (7.38)	21.98	18.89–27.65	- ^b^	-
SAM (μmol/L) (224) ^a^	0.91 (0.43)	0.85	0.62–1.00	- ^b^	-
SAH (μmol/L) (248) ^a^	0.88 (0.27)	0.83	0.69–1.00	- ^b^	-
Ratio SAM/SAH (224) ^a^	1.10 (0.59)	0.97	0.71–1.32	- ^b^	-
tHcy (μmol/L) (194) ^a^	2.71 (0.73)	2.66	2.27–3.12	<10 [56]	0%
Cystathionine (nmol/L) (194) ^a^	46.67 (28.22)	40.72	33.52–51.27	- ^b^	-
Cysteine (μmol/L) (194) ^a^	18.06 (15.78)	12.99	7.41–23.21	- ^b^	-
**B-vitamin-related Biomarkers (Laboratory)**					
Riboflavin (nmol/L) (218) ^a^	13.8 (9.4)	11.2	8.0–15.7	12.5–44.5 (10–18 years) [57]	62.9%
B2 subclass (nmol/L) (233) ^a^	63.7 (35.5)	57.3	47.2–71.5	- ^b^	-
Pyridoxal (nmol/L) (230) ^a^	8.6 (6.8)	7.7	6.1–9.4	8.8–58.7 (10–18 years) [58]	65.7%
PLP (nmol/L) (184) ^a^	39.1 (27.7)	32.4	24.1–44.8	20.5–151 (10–18 years) [58]	17.4%
B6 subclass (nmol/L) (233) ^a^	69.15 (90.3)	53.6	37.5–71.4	- ^b^	-
Plasma folate (ng/mL) (222) ^a^	4.9 (2.2)	4.6	3.4–6.2	15.6–16.6 (6–11 years) [59] 11.0–11.5 (12–19 years) [59]	98.6%
5-MTHF (nmol/L) (233) ^a^	22.9 (13.3)	21.1	11.8–30.5	26.4–219.7 (11–16 years) [60]	67.0%
Plasma B12 (pg/mL) (222) ^a^	435.5 (203.1)	396.0	301.2–527.0	713–743 (6–11 years) [59] 499–521 (12–19 years) [59]	77.5%

BMI, body mass index; MET, metabolic equivalent; FFQ, food frequency questionnaire; HEI, healthy eating index; LDL-c, low-density lipoprotein cholesterol; HDL-c, high-density lipoprotein cholesterol; ARA, arachidonic acid; EPA, eicosapentaenoic acid; DHA, docosahexaenoic acid; SFA, saturated fatty acids; MUFA, monounsaturated fatty acids; PUFA, polyunsaturated fatty acids; SAM, S-adenosylmethionine; SAH, S-adenosylhomocysteine; tHcy, total homocysteine; PLP, pyridoxal 5’-phosphate; 5-MTHF, 5-methyl tetrahydrofolate. ^a^ Values between brackets are the number of participants with available information for the biomarkers and the rest of variables used in the analyses. ^b^ There is no reference value for the age range of the study population.

**Table 2:** Association of lipid profile with B-vitamins biomarkers.

Dependent Variable (Log)	Independent Variable (Log)	β-Coefficients	*p*-Value ^a^	95% CI	R^2^
Total cholesterol		0.05	0.04 *	0.002; 0.11	0.07
Triglycerides		0.06	0.36	−0.07; 0.19	0.15
LDL-c	B2 subclass	0.09	0.02 *	0.01; 0.16	0.09
HDL-c		−0.006	0.83	−0.06; 0.05	0.17
Total cholesterol		0.05	<0.01 *	0.02; 0.09	0.09
Triglycerides		0.005	0.91	−0.08; 0.09	0.15
LDL-c	B6 subclass	0.06	0.02 *	0.01; 0.11	0.09
HDL-c		0.04	0.03 *	0.005; 0.08	0.19
Total cholesterol		0.01	0.59	−0.04; 0.07	0.06
Triglycerides		−0.03	0.63	−0.16; 0.10	0.15
LDL-c	Plasma folate	0.006	0.87	−0.07; 0.08	0.07
HDL-c		0.04	0.14	−0.01; 0.10	0.17
Total cholesterol		0.04	0.16	−0.02; 0.09	0.07
Triglycerides		−0.02	0.75	−0.16; 0.11	0.15
LDL-c	Plasma B12	0.05	0.20	−0.03; 0.13	0.08
HDL-c		0.03	0.33	−0.03; 0.09	0.18

CI, confidence interval; R^2^, coefficient of determination; LDL-c, low-density lipoprotein cholesterol; HDL-c, high-density lipoprotein cholesterol. ^a^ Multiple linear regression model, adjusted for sex, pubertal stage, BMI and HEI. * *p* < 0.05.

**Table 3:** Association of fatty acid profile with B-vitamins biomarkers.

Dependent Variable (Log)	Independent Variable (Log)	β-Coefficients	*p*-Value ^a^	95% CI	R^2^
15:0 pentadecanoic		−0.03	0.65	−0.16; 0.10	0.04
16:0 palmitic		0.01	0.33	−0.01; 0.04	0.02
16:1 (n-7) palmitoleic		0.12	<0.01 *	0.05; 0.20	0.17
18:0 stearic		0.001	0.94	−0.02; 0.03	0.01
18:1 (*trans*) elaidic		−0.10	<0.01 *	−0.16; −0.03	0.09
18:1 (n-9) oleic		0.06	<0.01 *	0.02; 0.10	0.07
18:2 (n-6) linoleic		0.15	<0.01 *	0.07; 0.24	0.14
18:3 (n-3) α−linolenic	B2 subclass	0.15	<0.01 *	0.05; 0.26	0.07
20:0 eicosanoic		−0.03	0.12	−0.08; 0.01	0.04
20:4 (n-6) ARA		0.20	<0.01 *	0.08; 0.33	0.12
20:5 (n-3) EPA		0.19	<0.01 *	0.06; 0.32	0.15
22:6 (n-3) DHA		0.25	<0.01 *	0.09; 0.41	0.12
SFA		0.009	0.50	−0.02; 0.04	0.01
MUFA		0.07	<0.01 *	0.03; 0.11	0.11
PUFA		0.19	<0.01 *	0.08; 0.29	0.13
n-3 PUFA		0.24	<0.01 *	0.09; 0.39	0.12
n-6 PUFA		0.18	<0.01 *	0.08; 0.28	0.13
Ratio n-6:n-3 PUFA		−0.06	0.08	−0.13; 0.008	0.10
15:0 pentadecanoic		0.09	0.04 *	0.002; 0.17	0.05
16:0 palmitic		0.008	0.40	−0.01; 0.03	0.01
16:1 (n-7) palmitoleic		0.03	0.29	−0.02; 0.08	0.13
18:0 stearic		0.007	0.44	−0.01; 0.02	0.01
18:1 (*trans*) elaidic		0.03	0.20	−0.02; 0.08	0.07
18:1 (n-9) oleic		0.002	0.91	−0.03; 0.03	0.04
18:2 (n-6) linoleic		−0.07	0.02 *	−0.12; −0.01	0.11
18:3 (n-3) α−linolenic	B6 subclass	−0.10	<0.01*	−0.18; −0.03	0.07
20:0 eicosanoic		0.02	0.10	−0.004; 0.05	0.04
20:4 (n-6) ARA		−0.10	0.02 *	−0.19; −0.02	0.10
20:5 (n-3) EPA		−0.05	0.28	−0.14; 0.04	0.12
22:6 (n-3) DHA		−0.12	0.03 *	−0.23; −0.01	0.10
SFA		0.003	0.72	−0.01; 0.02	0.01
MUFA		−0.01	0.37	−0.04; 0.02	0.07
PUFA		−0.09	0.02 *	−0.16; −0.02	0.11
n-3 PUFA		−0.11	0.03 *	−0.21; −0.01	0.10
n-6 PUFA		−0.08	0.02 *	−0.16; −0.02	0.10
Ratio n-6:n-3 PUFA		0.03	0.29	−0.02; 0.07	0.09
15:0 pentadecanoic		0.02	0.72	−0.11; 0.16	0.04
16:0 palmitic		−0.02	0.13	−0.05; 0.006	0.03
16:1 (n-7) palmitoleic		−0.05	0.20	−0.13; 0.03	0.14
18:0 stearic		−0.02	0.18	−0.05; 0.009	0.02
18:1 (*trans*) elaidic		−0.02	0.60	−0.09; 0.05	0.06
18:1 (n-9) oleic		0.02	0.50	−0.03; 0.06	0.04
18:2 (n-6) linoleic		0.15	<0.01 *	0.06; 0.23	0.13
18:3 (n-3) α−linolenic	Plasma folate	0.15	<0.01 *	0.04; 0.26	0.06
20:0 eicosanoic		−0.03	0.17	−0.07; 0.01	0.04
20:4 (n-6) ARA		0.22	<0.01 *	0.09; 0.35	0.11
20:5 (n-3) EPA		0.14	0.04 *	0.009; 0.28	0.13
22:6 (n-3) DHA		0.21	0.01 *	0.05; 0.38	0.11
SFA		−0.02	0.20	−0.05; 0.01	0.02
MUFA		0.03	0.22	−0.02; 0.07	0.07
PUFA		0.18	<0.01 *	0.07; 0.29	0.12
n-3 PUFA		0.20	0.01 *	0.05; 0.36	0.11
n-6 PUFA		0.18	<0.01 *	0.08; 0.29	0.12
Ratio n-6:n-3 PUFA		−0.02	0.66	−0.09; 0.06	0.09
15:0 pentadecanoic		0.10	0.18	−0.05; 0.24	0.05
16:0 palmitic		0.04	0.01 *	0.008; 0.07	0.04
16:1 (n-7) palmitoleic		0.13	<0.01 *	0.05; 0.22	0.17
18:0 stearic		0.05	<0.01 *	0.02; 0.08	0.06
18:1 (*trans*) elaidic		0.04	0.29	−0.03; 0.11	0.06
18:1 (n-9) oleic		0.06	<0.01 *	0.01; 0.11	0.07
18:2 (n-6) linoleic		−0.07	0.16	−0.16; 0.03	0.09
18:3 (n-3) α−linolenic	Plasma B12	−0.12	0.06	−0.23; 0.001	0.04
20:0 eicosanoic		0.05	0.02 *	0.009; 0.10	0.05
20:4 (n-6) ARA		−0.08	0.29	−0.22; 0.07	0.08
20:5 (n-3) EPA		−0.02	0.72	−0.17; 0.12	0.11
22:6 (n-3) DHA		−0.13	0.14	−0.31; 0.04	0.09
SFA		0.04	0.02 *	0.005; 0.06	0.03
MUFA		0.04	0.07	−0.004; 0.09	0.08
PUFA		−0.08	0.21	−0.20; 0.04	0.08
n-3 PUFA		−0.12	0.14	−0.29; 0.04	0.09
n-6 PUFA		−0.07	0.24	−0.19; 0.05	0.08
Ratio n-6:n-3 PUFA		0.06	0.17	−0.02; 0.13	0.10

CI, confidence interval; R^2^, coefficient of determination; ARA, arachidonic acid; EPA, eicosapentaenoic acid; DHA, docosahexaenoic acid; SFA, saturated fatty acids; MUFA, monounsaturated fatty acids; PUFA, polyunsaturated fatty acids. ^a^ Multiple linear regression model, adjusted for sex, pubertal stage, BMI and HEI. * *p* < 0.05.

**Table 4:** Association between 1C pathway metabolites with B-vitamins biomarkers.

Dependent Variable (Log)	Independent Variable (Log)	β-Coefficients	*p*-Value ^a^	95% CI	R^2^
Methionine		−0.02	0.68	−0.11; 0.07	0.02
SAM		−0.18	<0.01 *	−0.31; 0.04	0.08
SAH		0.01	0.79	−0.07; 0.09	0.02
SAM/SAH	B2 subclass	−0.20	<0.01 *	−0.34; −0.06	0.07
tHcy		−0.005	0.91	−0.10; 0.09	0.01
Cystathionine		−0.07	0.25	−0.21; 0.06	0.02
Cysteine		0.18	0.22	−0.11; 0.47	0.02
Methionine		0.002	0.95	−0.06; 0.06	0.02
SAM		0.12	0.01 *	0.03; 0.22	0.08
SAH		−0.03	0.34	−0.08; 0.03	0.03
SAM/SAH	B6 subclass	0.16	<0.01 *	0.06; 0.26	0.08
tHcy		−0.11	<0.01 *	−0.17; −0.05	0.08
Cystathionine		−0.06	0.15	−0.15; 0.02	0.02
Cysteine		−0.09	0.37	−0.28; 0.11	0.02
Methionine		0.01	0.79	−0.08; 0.11	0.02
SAM		0.11	0.13	−0.03; 0.26	0.05
SAH		−0.03	0.46	−0.12; 0.05	0.02
SAM/SAH	Plasma folate	0.13	0.11	−0.03; 0.28	0.04
tHcy		0.006	0.91	−0.09; 0.10	0.02
Cystathionine		0.07	0.30	−0.07; 0.21	0.02
Cysteine		0.16	0.31	−0.15; 0.47	0.02
Methionine		−0.08	0.11	−0.18; 0.02	0.04
SAM		0.09	0.25	−0.06; 0.24	0.05
SAH		−0.09	0.04 *	−0.18; −0.001	0.04
SAM/SAH	Plasma B12	0.17	0.04 *	0.01; 0.33	0.05
tHcy		−0.14	<0.01 *	−0.24; −0.04	0.06
Cystathionine		−0.20	<0.01 *	−0.33; −0.07	0.07
Cysteine		−0.06	0.72	−0.37; 0.25	0.02

CI, confidence interval; R^2^, coefficient of determination; SAM, S-adenosylmethionine; SAH, S-adenosylhomocysteine; tHcy, total homocysteine. ^a^ Multiple linear regression model, adjusted for sex, pubertal stage, BMI and HEI. * *p* < 0.05.

## Supplementary Materials

The following are available online at https://www.mdpi.com/2072-6643/11/12/2918/s1, Table S1. Association of lipid profile with B-vitamins biomarkers, Table S2. Association of fatty acid profile with B-vitamins biomarkers, Table S3. Association between 1C pathway metabolites with B-vitamins biomarkers, Table S4. Association of fatty acid profile with 1C pathway metabolites.
