# Supplementary material for: Vitamin B2 and Folate Concentrations are Associated with ARA, EPA and DHA Fatty Acids in Red Blood Cells of Brazilian Children and Adolescents
Source: Nutrients. 2019 Dec 2;11(12):2918. doi: 10.3390/nu11122918 (PMC6950420; doi:10.3390/nu11122918)
Supplement: Supplementary file 1 [file nutrients-11-02918-s001.pdf]

Supplementary Material

Table S1. Association of lipid profile with B-vitamins biomarkers.

Dependent variable (log)	Independent variable (log)	β-coefficients	p-value[a]	95% CI	R^2
Total cholesterol		0.03	0.15	-0.01 ; 0.08	0.08
Triglycerides		0.06	0.33	-0.06 ; 0.17	0.14
LDL-c	Riboflavin	0.06	0.07	-0.005 ; 0.12	0.09
HDL-c		-0.01	0.59	-0.06 ; 0.04	0.18
Total cholesterol		0.04	0.21	-0.02 ; 0.10	0.07
Triglycerides		-0.03	0.70	-0.17 ; 0.11	0.15
LDL-c	Pyridoxal	0.04	0.28	-0.04 ; 0.12	0.07
HDL-c		0.03	0.33	-0.03 ; 0.09	0.18
Total cholesterol		0.03	0.19	-0.02 ; 0.08	0.07
Triglycerides		-0.05	0.45	-0.17 ; 0.08	0.14
LDL-c	PLP	0.04	0.30	-0.03 ; 0.11	0.08
HDL-c		0.04	0.12	-0.01 ; 0.09	0.20
Total cholesterol		0.04	0.06	-0.002 ; 0.07	0.07
Triglycerides		0.04	0.38	-0.05 ; 0.13	0.15
LDL-c	5-MTHF	0.05	0.05	0.001 ; 0.10	0.08
HDL-c		-0.002	0.92	-0.04 ; 0.04	0.17

CI, confidence interval; R^2, coefficient of determination; PLP, pyridoxal 5'-phosphate; 5-MTHF, 5-methyl tetrahydrofolate; LDL-c, low-density lipoprotein cholesterol; HDL-c, high-density lipoprotein cholesterol. [a] Multiple linear regression model, adjusted for sex, pubertal stage, BMI and HEI. *p<0.05.

Table S2. Association of fatty acid profile with B-vitamins biomarkers.

Dependent variable (log)	Independent variable (log)	β-coefficients	p-value[a]	95% CI	R^2
15:0 pentadecanoic		0.03	0.57	-0.08 ; 0.15	0.05
16:0 palmitic		0.02	0.18	-0.007 ; 0.04	0.02
16:1 (*n-7*) palmitoleic		0.07	0.04*	0.004 ; 0.14	0.14
18:0 stearic		0.009	0.42	-0.01 ; 0.03	0.01
18:1 (*trans*) elaidic		0.02	0.61	-0.04 ; 0.07	0.06
18:1 (*n-9*) oleic		0.02	0.38	-0.02 ; 0.05	0.05
18:2 (*n-6*) linoleic		-0.02	0.62	-0.09 ; 0.05	0.10
18:3 (*n-3*) α-linolenic	Riboflavin	0.008	0.88	-0.09 ; 0.10	0.04
20:0 eicosanoic		0.002	0.58	-0.04 ; 0.04	0.03
20:4 (*n-6*) ARA		-0.02	0.72	-0.14 ; 0.10	0.08
20:5 (*n-3*) EPA		0.07	0.27	-0.05 ; 0.19	0.12
22:6 (*n-3*) DHA		0.03	0.69	-0.12 ; 0.18	0.08
SFA		0.01	0.31	-0.01 ; 0.04	0.01
MUFA		0.01	0.51	-0.03 ; 0.05	0.07
PUFA		-0.01	0.78	-0.11 ; 0.08	0.09
n-3 PUFA		0.03	0.65	-0.11 ; 0.17	0.09
n-6 PUFA		-0.02	0.69	-0.11 ; 0.07	0.09
Ratio *n-6*:*n-3* PUFA		-0.05	0.12	-0.12 ; 0.01	0.10
15:0 pentadecanoic		0.06	0.39	-0.08 ; 0.20	0.04

16:0 palmitic		0.02	0.14	-0.007 ; 0.05	0.02
16:1 (*n-7*) palmitoleic		0.07	0.11	-0.02 ; 0.16	0.14
18:0 stearic		0.02	0.27	-0.01 ; 0.05	0.01
18:1 (*trans*) elaidic		-0.05	0.16	-0.13 ; 0.02	0.07
18:1 (*n-9*) oleic		0.002	0.92	-0.04 ; 0.05	0.04
18:2 (*n-6*) linoleic		-0.07	0.14	-0.17 ; 0.02	0.10
18:3 (*n-3*) α-linolenic	Pyridoxal	-0.19	< 0.01*	-0.30 ; -0.07	0.08
20:0 eicosanoic		0.03	0.16	-0.01 ; 0.08	0.03
20:4 (*n-6*) ARA		-0.12	0.11	-0.27 ; 0.03	0.09
20:5 (*n-3*) EPA		-0.06	0.41	-0.21 ; 0.09	0.13
22:6 (*n-3*) DHA		-0.14	0.13	-0.32 ; 0.04	0.09
SFA		0.01	0.36	-0.02 ; 0.05	0.01
MUFA		-0.02	0.52	-0.06 ; 0.03	0.06
PUFA		-0.10	0.10	-0.22 ; 0.02	0.10
n-3 PUFA		-0.14	0.12	-0.31 ; 0.03	0.09
n-6 PUFA		-0.10	0.11	-0.21 ; 0.02	0.09
Ratio *n-6*:*n-3* PUFA		0.04	0.34	-0.04; 0.12	0.09
15:0 pentadecanoic		0.09	0.16	-0.04 ; 0.22	0.06
16:0 palmitic		0.001	0.93	-0.03 ; 0.03	0.02
16:1 (*n-7*) palmitoleic		0.11	< 0.01*	0.03 ; 0.18	0.13
18:0 stearic		-0.003	0.85	-0.03 ; 0.02	0.02
18:1 (*trans*) elaidic		-0.006	0.85	-0.08 ; 0.06	0.05
18:1 (*n-9*) oleic		0.02	0.44	-0.03 ; 0.06	0.04
18:2 (*n-6*) linoleic		-0.02	0.68	-0.10 ; 0.07	0.09
18:3 (*n-3*) α-linolenic	PLP	-0.07	0.21	-0.17 ; 0.04	0.05
20:0 eicosanoic		0.02	0.47	-0.03 ; 0.06	0.05
20:4 (*n-6*) ARA		-0.009	0.89	-0.14 ; 0.12	0.07
20:5 (*n-3*) EPA		0.11	0.10	-0.02 ; 0.23	0.13
22:6 (*n-3*) DHA		0.04	0.61	-0.12 ; 0.20	0.07
SFA		0.005	0.73	-0.02 ; 0.03	0.01
MUFA		0.02	0.49	-0.03 ; 0.06	0.06
PUFA		-0.009	0.88	-0.12 ; 0.10	0.08
n-3 PUFA		0.04	0.57	-0.11 ; 0.19	0.07
n-6 PUFA		-0.01	0.79	-0.12 ; 0.09	0.08
Ratio *n-6*:*n-3* PUFA		-0.06	0.10	-0.13 ; 0.01	0.07
15:0 pentadecanoic		-0.03	0.49	-0.12 ; 0.06	0.04
16:0 palmitic		0.001	0.99	-0.02 ; 0.02	0.01
16:1 (*n-7*) palmitoleic		0.001	0.97	-0.06 ; 0.06	0.13
18:0 stearic		-0.03	0.74	-0.02 ; 0.01	0.01
18:1 (*trans*) elaidic		-0.04	0.83	-0.09 ; 0.006	0.07
18:1 (*n-9*) oleic		0.009	0.54	-0.02 ; 0.04	0.04
18:2 (*n-6*) linoleic		0.02	0.49	-0.04 ; 0.08	0.09
18:3 (*n-3*) α-linolenic	5-MTHF	0.02	0.53	-0.05 ; 0.10	0.03
20:0 eicosanoic		0.006	0.67	-0.02 ; 0.04	0.03
20:4 (*n-6*) ARA		0.03	0.59	-0.07 ; 0.12	0.08
20:5 (*n-3*) EPA		-0.02	0.73	-0.11 ; 0.08	0.12
22:6 (*n-3*) DHA		0.01	0.84	-0.11 ; 0.13	0.08
SFA		-0.001	0.91	-0.02 ; 0.02	0.01
MUFA		0.007	0.63	-0.02 ; 0.04	0.06
PUFA		0.02	0.62	-0.06 ; 0.10	0.08
n-3 PUFA		0.007	0.89	-0.10 ; 0.12	0.08

n-6 PUFA		0.02	0.56	-0.05 ; 0.10	0.08
Ratio *n-6*:*n-3* PUFA		0.02	0.53	-0.04 ; 0.07	0.09

CI, confidence interval; R^2, coefficient of determination; PLP, pyridoxal 5'-phosphate; 5-MTHF, 5-methyl tetrahydrofolate; ARA, arachidonic acid; EPA, eicosapentaenoic acid; DHA, docosahexaenoic acid; SFA, saturated fatty acids; MUFA, monounsaturated fatty acids; PUFA, polyunsaturated fatty acids. [a] Multiple linear regression model, adjusted for sex, pubertal stage, BMI and HEI. *p<0.05.

Table S3. Association between 1C pathway metabolites with B-vitamins biomarkers.

Dependent variable (log)	Independent variable (log)	β-coefficients	p-value[a]	95% CI	R^2
Methionine		-0.02	0.71	-0.09 ; 0.06	0.01
SAM		-0.22	< 0.01*	-0.34 ; -0.09	0.10
SAH		-0.01	0.74	-0.09 ; 0.06	0.02
SAM/SAH	Riboflavin	-0.21	< 0.01*	-0.34 ; -0.08	0.08
tHcy		-0.001	0.98	-0.08 ; 0.08	0.03
Cystathionine		-0.01	0.86	-0.12 ; 0.10	0.02
Cysteine		-0.15	0.25	-0.39 ; 0.10	0.04
Methionine		0.11	0.04*	0.006 ; 0.21	0.04
SAM		0.09	0.24	-0.06 ; 0.25	0.06
SAH		0.03	0.57	-0.07 ; 0.12	0.02
SAM/SAH	Pyridoxal	0.08	0.34	-0.08 ; 0.24	0.04
tHcy		-0.07	0.16	-0.17 ; 0.03	0.02
Cystathionine		-0.15	0.04*	-0.29 ; -0.01	0.04
Cysteine		0.28	0.08	-0.04 ; 0.59	0.03
Methionine		0.04	0.40	-0.06 ; 0.14	0.04
SAM		0.07	0.38	-0.07 ; 0.21	0.05
SAH		-0.02	0.68	-0.10 ; 0.07	0.03
SAM/SAH	PLP	0.09	0.22	-0.06 ; 0.24	0.03
tHcy		-0.06	0.15	-0.15 ; 0.02	0.05
Cystathionine		0.01	0.87	-0.12 ; 0.14	0.01
Cysteine		0.15	0.31	-0.14 ; 0.44	0.03
Methionine		0.06	0.11	-0.01 ; 0.13	0.03
SAM		-0.03	0.55	-0.13 ; 0.07	0.05
SAH		0.005	0.87	-0.05 ; 0.06	0.02
SAM/SAH	5-MTHF	-0.04	0.49	-0.15 ; 0.07	0.04
tHcy		-0.01	0.71	-0.08 ; 0.05	0.01
Cystathionine		-0.03	0.51	-0.13 ; 0.06	0.02
Cysteine		0.17	0.12	-0.05 ; 0.38	0.03

CI, confidence interval; R^2, coefficient of determination; PLP, pyridoxal 5'-phosphate; 5-MTHF, 5-methyl tetrahydrofolate; SAM, S-adenosylmethionine; SAH, S-adenosylhomocysteine; tHcy, total homocysteine. [a] Multiple linear regression model, adjusted for sex, pubertal stage, BMI and HEI. *p<0.05.

Table S4. Association of fatty acid profile with 1C pathway metabolites.

Dependent variable (log)	Independent variable (log)	β-Coefficients	p-value[a]	95% CI	R^2
18:2 (*n-6*) linoleic		-0.04	0.35	-0.12 ; 0.04	0.12
18:3 (*n-3*) α-linolenic		-0.05	0.34	-0.16 ; 0.06	0.05
20:4 (*n-6*) ARA		0.003	0.96	-0.13 ; 0.13	0.09

20:5 (*n-3*) EPA		0.02	0.77	-0.11 ; 0.15	0.14
22:6 (*n-3*) DHA	SAM	0.001	0.99	-0.16 ; 0.16	0.08
SFA		-0.02	0.07	-0.05 ; 0.003	0.03
MUFA		-0.006	0.77	-0.05 ; 0.04	0.07
PUFA		-0.02	0.79	-0.13 ; 0.09	0.10
n-3 PUFA		-0.001	0.99	-0.15 ; 0.15	0.09
n-6 PUFA		-0.02	0.76	-0.12 ; 0.09	0.10
18:2 (*n-6*) linoleic		0.05	0.44	-0.08 ; 0.19	0.10
18:3 (*n-3*) α-linolenic		0.01	0.88	-0.16 ; 0.18	0.04
20:4 (*n-6*) ARA		0.12	0.28	-0.09 ; 0.32	0.09
20:5 (*n-3*) EPA		0.09	0.38	-0.11 ; 0.30	0.13
22:6 (*n-3*) DHA	SAH	0.27	0.04*	0.01 ; 0.52	0.09
SFA		-0.004	0.86	-0.05 ; 0.04	0.01
MUFA		0.04	0.21	-0.02 ; 0.11	0.06
PUFA		0.10	0.26	-0.07 ; 0.27	0.09
n-3 PUFA		0.24	0.05	-0.002 ; 0.48	0.09
n-6 PUFA		0.08	0.32	-0.08 ; 0.25	0.09
18:2 (*n-6*) linoleic		-0.07	0.08	-0.15 ; 0.01	0.12
18:3 (*n-3*) α-linolenic		-0.07	0.19	-0.17 ; 0.03	0.05
20:4 (*n-6*) ARA		-0.06	0.35	-0.18 ; 0.06	0.09
20:5 (*n-3*) EPA		-0.04	0.56	-0.16 ; 0.09	0.14
22:6 (*n-3*) DHA	SAM/SAH	-0.12	0.11	-0.27 ; 0.03	0.10
SFA		-0.02	0.08	-0.05 ; 0.003	0.03
MUFA		-0.03	0.16	-0.07 ; 0.01	0.08
PUFA		-0.07	0.19	-0.17 ; 0.04	0.11
n-3 PUFA		-0.11	0.13	-0.25 ; 0.03	0.10
n-6 PUFA		-0.06	0.21	-0.16 ; 0.04	0.10
18:2 (*n-6*) linoleic		0.24	< 0.01*	0.09 ; 0.38	0.14
18:3 (*n-3*) α-linolenic		0.24	0.02*	0.05 ; 0.43	0.07
20:4 (*n-6*) ARA		0.38	< 0.01*	0.16 ; 0.60	0.12
20:5 (*n-3*) EPA		0.35	< 0.01*	0.13 ; 0.57	0.19
22:6 (*n-3*) DHA	Homocysteine	0.49	< 0.01*	0.22 ; 0.75	0.13
SFA		0.01	0.60	-0.03 ; 0.06	0.01
MUFA		0.09	0.03*	0.009 ; 0.16	0.08
PUFA		0.33	< 0.01*	0.15 ; 0.51	0.14
n-3 PUFA		0.46	< 0.01*	0.21 ; 0.71	0.13
n-6 PUFA		0.31	< 0.01*	0.13 ; 0.49	0.13

CI, confidence interval; R^2, coefficient of determination; PLP, pyridoxal 5'-phosphate; 5-MTHF, 5-methyl tetrahydrofolate; SAM, S-adenosylmethionine; SAH, S-adenosylhomocysteine; SFA, saturated fatty acids; MUFA, monounsaturated fatty acids; PUFA, polyunsaturated fatty acids. [a] Multiple linear regression model, adjusted for sex, pubertal stage, BMI and HEI. *p<0.05.
